# Effects of Perceived Accessibility to Living Infrastructure on Positive Feelings Among Older Adults

**DOI:** 10.3390/bs14111025

**Published:** 2024-11-01

**Authors:** Sohee Kim

**Affiliations:** Department of Social Welfare and Child Studies, Daejin University, Pocheon-si 11159, Republic of Korea; niki88@daejin.ac.kr

**Keywords:** perceived accessibility to living infrastructure, motility, life satisfaction, happiness, social participation, older adults

## Abstract

Social participation among older adults is a critical aspect that facilitates the improvement of their overall well-being. A critical factor influencing the social participation of older adults to achieve optimal aging is perceived accessibility to living infrastructure. The study aims to provide a comprehensive analysis of how perceived accessibility to transportation systems, public service facilities, and digital services influences life satisfaction and happiness among older adults. Survey data were collected from 200 households in South Korea and the research paper utilized the Partial Least Squares (PLSs) bootstrapping methodology with 5000 subsample iterations for analysis. The study shows that perceived accessibility to transportation systems, public service facilities, and digital services significantly influenced satisfaction among older adults. Satisfaction, in turn, had a positive effect on happiness. The implications for theory and practical implications were provided for officials and social service professionals concerning the geriatric population.

## 1. Introduction

The topics of accessibility and inclusion of older adults are emerging as vital global challenges, which are foreseen to increase in importance in the upcoming years [1]. The term “old age” denotes a phase in the human lifespan characterized by a decline in physical strength and functional capacity, attributable to alterations in the cellular composition of the body [2]. Age-related factors, including physical constraints and driving cessation, often result in a decline in involvement in social activities in the later life stages. Furthermore, the global demographic is experiencing rapid aging, with the population segment aged over 60 expected to exceed the 1 billion threshold within the next decade [1].

Due to population aging, the investigation of factors that promote improvements in the health and well-being of older adults has become the primary concern in most countries around the world [3,4]. In older age, individuals’ psychophysical health is considerably influenced by a variety of determinants, including their levels of physical activity, social participation, and perceptions about aging [5].

The demographic structure of South Korea has undergone rapid changes over the last 70 years, with the proportion of individuals aged 65 and above rising from under 3 percent in 1950 to 15 percent currently and the United Nations forecasting that this demographic will constitute 40 percent by the mid-2060s [6]. The country presently features one of the highest life expectancies worldwide, with infants predicted to live, on average, for 82 years (79 for males, 85 for females) and with the UN projecting a further rise in life expectancy in Korea [7]. In the 2018 Seoul Aged Survey, 86.3% of older respondents anticipated that at least half of the health they currently possessed preferred to remain in their existing residences [8]. Aging-in-place refers to an approach that helps older adults to remain in their residences for an extended duration [9]. The livability of older adults is significantly shaped by their social and built environments. The built environment includes commodities that are designed and altered by people such as housing, transportation, and the attributes of local communities [10]. Thus, infrastructure and services should be modified to ensure accessibility and inclusivity for older adults with diverse needs, thereby facilitating their safe and suitable aging in place [6]. Aging-in-place signifies and strengthens an affinity toward one’s residence and the surrounding community, leading to enhanced social participation as a consequence [11].

Social participation, defined as *“a person’s involvement in social activities that provide social interactions within his/her community or society”* [12], is a key component within aging theories [13,14]. The previous literature has provided empirical evidence indicating that social participation is associated with advancements in physical health [15], a decrease in cognitive decline [16], and improvements in quality of life [17]. Furthermore, social participation is related to an augmented capacity for individuals to engage in independent living at home for prolonged periods [18]. Thus, social participation among older adults is a critical aspect that facilitates the improvement in their overall well-being [19].

As we gain a better understanding of the value of social participation of older adults, there is growing interest in the role of the built environment [20]. Previous studies have reported positive relationships between social factors and the overall well-being of older adults. Older adults can engage in active aging within a community that maximizes “opportunities for health, participation and security in order to enhance quality of life as people age”, with the World Health Organization’s age-friendliness framework, which is increasingly integrated into local and regional policies [21]. Recently, rather than social factors, scholarly focus has shifted toward the built environment and its relationships with the well-being of older adults, particularly through the lens of aging-in-place [22]. Little attention has been given to the built environment and older adults who wish to age in place while actively participating in their communities. Furthermore, even fewer pieces of research have been undertaken to thoroughly investigate the effects of the accessibility to living infrastructure on positive feelings among older adults.

A critical factor influencing the social participation of older adults to achieve optimal aging is the accessibility of the services and facilities available within their living environment [23,24]. Accessibility, from the perspective of social infrastructure, is a multifaceted concept that encompasses the ease with which individuals can access essential services and facilities, which are crucial for their daily lives and overall well-being [25]. In the context of social infrastructure, accessibility is often measured by the travel time required for residents to reach various essential services such as supermarkets, bus stops, primary schools, and healthcare facilities. The need for accessible and affordable transportation, information about available activities, and adapted social opportunities is paramount to fostering active social participation [26].

However, although the concept of accessibility is not only about transportation accessibility but also involves socio-economic and equity considerations, previous research has focused on transportation accessibility [27,28,29]. The extant accessibility measures are insufficient in encapsulating all the factors that affect social participation and well-being among older adults. Consequently, it is necessary to expand the accessibility in terms of living infrastructure, affecting the promotion of social participation among older adults.

### 1.1. Perceived Accessibility to Living Infrastructure

Living infrastructure reflects equal accessibility [30]. In contrast to the notion of social infrastructure, which is delineated as the tangible environment that facilitates the effective cultivation of social capital [31], living infrastructure pertains to a dynamic and accessible infrastructure that residents can readily engage with and utilize through both offline and online modalities [32]. For example, the notion of living infrastructure includes educational institutions, libraries, facilities catering to older adults, healthcare clinics, recreational sports centers, commercial retail establishments, and transportation services [33]. 

The scholarly discussion on aging-in-place focuses on the vital role of living infrastructure within the context of community. There exists an increasing scholarly interest in the concept of community-based social capital and its ramifications on the phenomenon of aging in place [34]. In this context, social capital can be defined as a particular kind of capital that reveals itself through informal exchanges with peers and dedicated participation in community life from an individualistic lens [35]. This concept implies that living infrastructure may be profoundly associated with social capital, as strong community ties and supportive frameworks can significantly improve the living conditions and resources accessible to older adults. Thus, improving social capital could indirectly yield living infrastructure, facilitating a more favorable environment for aging in place [36].

The deployment of living infrastructure has the ability to improve the comfort of everyday life and enhance welfare by supporting the regular activities of individuals. Consequently, this subsequently influences the overall quality of life and subjective well-being among older adults [37]. Living infrastructure significantly impacts individual subjective well-being by fostering social cohesion and interpersonal connections, thereby augmenting social participation and facilitating opportunities for social interactions [38].

Furthermore, motility, which is defined as the capacity of entities (e.g., goods, information, or persons) to be mobile in social and geographic space [39] allows for more holistic explanatory models with regard to accessibility to living infrastructure. While spatial mobility clearly represents the physical movement of individuals across geographical territories, motility has been both conceptually and operationally framed to include the concepts of resources and potential. Motility, interpreted as the ‘potential to engage in travel’, incorporates the diverse personal resources required for mobility and illustrates the accumulated significance of mobility experiences alongside the role of mobility in broadening the availability of opportunities [40]. The interplay between motility and mobility is complex, complicating the task of discerning the directionality of their influence due to its inherently cyclic nature: motility promotes mobility experiences, which in turn enhances motility and increases potential mobility. Access, one of the elements of motility, refers to the spectrum of potential mobilities determined by spatial, temporal, and various contextual constraints. Access is restricted by options and conditions. The options include the entire range of transportation and communication modalities that exist, as well as the total set of services and equipment accessible at a particular instant. The conditions relate to the availability of these options, assessed in terms of location-specific expenses, logistical factors, and other relevant limitations.

According to the theory of motility, Yun and Noh [41] explicitly outline three dimensions of accessibility to mobilities, including accessibility to transportation systems, accessibility to public service facilities, and accessibility to digital services. It emphasizes the importance of accessibility in promoting social participation [39]. First, accessibility to transportation systems is fundamentally associated with public transit systems and includes parameters that assess the level of user convenience. Second, accessibility to public service facilities is determined by the assessment of daily living resources within the local community and functions as a standard for evaluating accessibility. The variables evaluated encompass accessibility to administrative agencies, as well as significant facilities such as healthcare institutions, cultural and welfare establishments, and essential living resources, in addition to educational institutions, all of which are pertinent to the evaluation of accessibility. Finally, accessibility to digital services is related to the interaction with critical data necessary for navigation and assesses the scope of engagement with information relevant to daily activities. The consistent variables in this context consist of the web-based utilization of public transport resources (through mobile devices or PCs), access to traffic information via online platforms, digital banking transactions, interactions with business and governmental agencies, etc.

Meanwhile, the perceived accessibility of living infrastructure serves as a more significant determinant than alternative objective metrics, such as the quantity of available facilities and spatial area, in assessing the satisfaction derived from living infrastructure and the propensity for utilization among the older adult population [33,42]. Consequently, within the framework of this research, perceived accessibility to living infrastructure was evaluated employing the perceived accessibility scale (PAC). The PAC scale is fundamentally based on subjective perceptions and the relative ease of access to living infrastructure, through the implementation of the measurement methodology articulated by Lättman et al. [43].

Thus, the study aims to examines the condition of perceived accessibility to living infrastructure, including accessibility to transportation systems, accessibility to public service facilities, and accessibility to digital services and to investigate the relationship between perceived accessibility to living infrastructure and subjective well-being among older adults. As subjective wellbeing, life satisfaction can be defined as a person’s self-assessed satisfaction with their life across multiple domains including health and social relationships [44].

#### 1.1.1. Perceived Accessibility to Transportation Systems

Previous studies have underscored the importance of mobility in influencing the quality of life and overall wellbeing in the later phases of life [45,46]. Clearly, mobility is crucial to the notion of active aging and aging-in-place, and it is profoundly associated with health status and enhanced subjective well-being [47]. Mobility is influenced by perceived accessibility to transportation systems, with barriers such as time, economic resources, and the geographical context affecting individuals’ ability to utilize public transport effectively. Perceived accessibility to transportation systems refers to the possibility of going somewhere to reach the activities of preference [48]. It enables older adults to engage with others in various activities, which subsequently mitigates social exclusion [49] and enhances overall well-being [50]. Transport inclusion, which aims to create accessible transport systems, is another critical factor influencing life satisfaction among older adults. It enhances older adults’ health, social participation, and subjective well-being by improving opportunity accessibility, physical accessibility, age-friendly informationalization, and cost affordability [27]. This relationship affects health, social participation, and subjective well-being. It emphasizes the importance of perceived accessibility to transportation systems in enhancing life satisfaction among older adults [51]. Thus, higher accessibility to transportation systems will likely lead to greater life satisfaction among adults. This study posits the following subsequent research hypotheses:

**Hypothesis (H1).**  *Perceived accessibility to transportation systems positively influences life satisfaction.*

#### 1.1.2. Perceived Accessibility to Public Service Facilities

Theoretically, aging-in-place pertains to a residential environment that facilitates the autonomy of older adults residing within the community [52]. This encompasses the establishment of a sustainable living environment. An accessible living environment is of great importance for older adults to enable their transition away from their living spaces. A pivotal indicator of a sustainable built environment is the notion of perceived accessibility to public service facilities. It refers to the relative accessibility for contact or use of services [53]. Numerous factors motivate older adults to leave their residences, encompassing avenues for acquiring novel knowledge, engagement in physical activity, and access to various services such as older adult day programs, healthcare facilities, or fitness centers [54]. A primary outcome of perceived accessibility to public service facilities is the enhancement of social engagement and the improvement in general health outcomes [55]. Furthermore, perceived accessibility to public service facilities significantly contributes to psychological well-being [56], and this relationship is mediated by increased physical activity and a stronger sense of community belonging [57]. Thus, higher accessibility to public service facilities will likely lead to greater life satisfaction among adults [58]. This study suggests the following research hypotheses:

**Hypothesis (H2).**  *Perceived accessibility to public service facilities positively influences life satisfaction.*

#### 1.1.3. Perceived Accessibility to Digital Services

Perceived accessibility to digital services refers to the ease with which digital services are reached. It can be characterized as the ease of use that enables older adults to execute tasks with efficacy and efficiency while simultaneously deriving satisfaction from the experience [59]. While digital technologies offer opportunities for enhanced social participation, they also pose challenges for older adults who may lack the necessary skills or resources to engage effectively. Digital literacy refers to “the ability to use information and communication technologies to find, evaluate, create, and communicate information” [60]. The lack of digital literacy in older adults can lead to social exclusion, particularly for those in residential facilities [61,62]. Digital literacy empowers older adults to mitigate feelings of isolation, maintain interpersonal connections, and engage in a diverse array of activities [63]. Digital literacy, in particular, had the strongest impact on life satisfaction, suggesting that perceived accessibility to digital services is crucial for enhancing life satisfaction among older adults. Digital accessibility is another critical aspect, especially in the context of increasing digital literacy, which in turn develops higher life satisfaction among older adults. In South Korea, a study found that perceived accessibility to digital services was consistently linked to higher life satisfaction among older adults from 2019 to 2022 [64]. Thus, higher perceived accessibility to digital services will likely lead to greater life satisfaction among adults. This study proposes the following research hypotheses:

**Hypothesis (H3).**  *Perceived accessibility to digital services positively influences life satisfaction.*

### 1.2. Life Satisfaction and Happiness

Life satisfaction refers to a reflective assessment of life quality, influenced by personal aspirations and achievements [65]. It exhibits greater stability and may fluctuate according to varying life situations. Conversely, happiness is frequently regarded as a more immediate indicator of subjective well-being, embodying transient emotional experiences [66]. Although both constructs are interconnected, they fulfill distinct functions in the comprehension of well-being, with life satisfaction offering a holistic life perspective and happiness encapsulating ephemeral emotional states.

Life satisfaction plays a crucial role in influencing happiness levels among older adults, as evidenced by multiple studies examining various factors that contribute to life satisfaction and its subsequent impact on happiness. The study by Kim [67] highlights that life satisfaction acts as a mediating factor between social participation and happiness. This result suggests that higher life satisfaction can enhance happiness among older adults. Other studies have demonstrated a positive relationship between life satisfaction and happiness, indicating that higher life satisfaction leads to greater happiness among older adults [68]. Similarly, Kolosnitsyna et al. [69] demonstrated a positive relationship between social support, life satisfaction, and happiness. The work indicates that older adults with higher life satisfaction and social support from family and friends tend to experience greater happiness. Thus, higher life satisfaction will likely lead to greater happiness among adults. This study proposes the following research hypotheses:

**Hypothesis (H4).**  *Life satisfaction positively influences happiness.*

**Figure 1:**
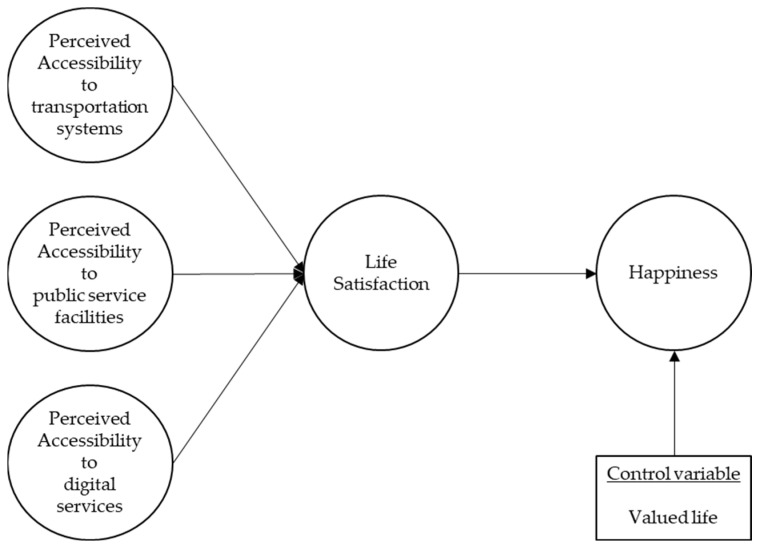
Research model.

This study also included valued life as a control variable positively affecting happiness. Meaning in life pertains to the perception individuals possess regarding the coherence and significance of their existence and experiences. A valued life of a meaningful life measures a sense of life’s inherent value [70]. Individuals who experience this phenomenon, characterized by a pronounced sense of meaning in life, report elevated levels of happiness [71]. Collectively, the integration of the existing literature has led to the development of the research model depicted in Figure 1.

## 2. Materials and Methods

### 2.1. Participants and Procedure

Because the primary purpose of this study is to explore the types of perceived accessibility to living infrastructure and to investigate its consequences on positive feelings among older adults, this research project encompassed a demographic of older adults aged 60 years and over those residing across the Republic of Korea. 

Conducting a minor pilot study or exploratory investigation utilizing non-probability sampling techniques may provide valuable insights. Thus, a sampling methodology based on non-probability principles was utilized for data collection. The data collection process was executed by a firm proficient in online research methodologies, employing a mobile application to facilitate an open survey panel. A total of 220 respondents were involved in the survey, out of which a curated sample of 200 (90.9%) was utilized for the ultimate analysis of this investigation after the elimination of dubious responses.

Table 1 delineates the characteristics of the respondents who engaged in this empirical study. The sample comprises 95 males (47.5%) and 105 females (52.5%). The age demographics reveal that 55 individuals fall within the 65 to 69 age range (27.5%), 95 individuals are aged between 70 and 74 (47.5%), and 50 individuals are aged 75 and above (25.0%). In terms of educational attainment, the sample includes 90 individuals who are high school graduates or below (45.0%), 94 college graduates (47.0%), and 16 individuals who have completed graduate studies (8.0%). The respondents’ residential distribution indicates that 59 (29.5%) reside in Seoul City; 78 (39.0%) are from the metropolitan area surrounding Seoul; 33 (16.5%) originate from other major metropolitan cities; and 30 (15.0%) are from other regions. Furthermore, the demographic analysis of the average monthly income (in KRW) indicates that 11 individuals report earnings of two million or less (5.5%), 53 individuals receive an income ranging from two million to three million (26.5%), 101 individuals earn between three million and four million (50.5%), 31 individuals have earnings that fall between four million and five million (15.5%), and 4 individuals earn five million or more (2.0%).

### 2.2. Instruments

The constructs of the research were measured utilizing established measurements derived from previous research, which were modified to align with the specific research context through minimal rephrasing. All measurement scales were operationalized as reflective constructs and evaluated employing five-point Likert scales. Excluding happiness, the Likert-type evaluations ranged from 1 (strongly disagree) to 5 (strongly agree). The specific instruments employed for the assessment of each construct, along with their respective sources, are detailed in Appendix A. 

As a dependent variable, perceived accessibility to transportation systems was measured using items from Lättman et al. [43] and Yun [72]. Perceived accessibility to public service facilities was the modification of those developed by Kim et al. [73] and Watthanaklang et al. [74]. Measures of perceived accessibility to digital services were drawn from Lättman et al. [43] and Yun [72]. 

For the assessment of positive feelings among older adults, the measurement of satisfaction was the modification of those developed by Diener et al. [75]. The items used for happiness were developed by Lyubomirsky and Lepper [76]. As a control variable, the items for valued life were adapted from Morgan and Farsides [70]. A multitude of demographic variables was incorporated into the study: sex, chronological age, educational attainment, and mean monthly earnings.

As a result of gathering data concerning both independent and dependent variables via a survey, the variance inflation factor (VIF) values were analyzed to assess the existence of common method bias (CMB) within our sample [77]. The outcomes of the analysis indicated that the VIF values fluctuated from 1.052 to 2.220, significantly below the suggested threshold of 3.3 [78], thereby implying that CMB did not pose an issue for the data (refer to Appendix B).

## 3. Results

This study utilized the Partial Least Squares (PLSs) methodology to evaluate the proposed research framework. In addition to the benefits inherent in the PLS approach, including its capacity to handle non-normally distributed data and the incorporation of formatively assessed latent constructs, the author selected PLS analysis due to its efficacy with limited sample sizes [79]. Given that the principal aim of this study was to predict outcomes rather than to validate a pre-existing theoretical framework, PLS analysis was deemed an appropriate choice for this research [80].

### 3.1. Measurement Model Assessment

The research conducted the reliability and validity of the measurements [81]. For construct reliability, as evidenced by the data presented in Table 2, Cronbach’s alpha coefficients achieved the minimum requisite threshold of 0.7, while all composite reliability (CR) indices surpassed the recommended threshold of 0.7 [82,83].

For the assessment of convergent validity, as illustrated in Appendix C, each individual item exhibited substantial loadings on their designated constructs. The factor loadings varied from 0.725 to 0.930. Furthermore, as demonstrated in Table 2, the Average Variance Extracted (AVE) across all constructs surpassed the threshold of 0.5 [82,83].

In the context of assessing discriminant validity, the correlation coefficient between the specific indicator and its corresponding construct was observed to exceed the correlation coefficients with alternative block constructs, thereby evidencing the presence of discriminant validity (refer to Appendix C). Furthermore, it is imperative that the square root of the Average Variance Extracted (AVE) exceeds the inter-construct correlation values [83]. As illustrated by the inter-construct correlations (off-diagonal elements) and the square root of the Average Variance Extracted (AVE) (diagonal elements) presented in Table 3, the diagonal elements of the correlation matrix surpassed the associated correlations with other constructs. Furthermore, the researcher evaluated the heterotrait–monotrait ratio of correlation (HTMT) methodology [84]. As delineated in Appendix D, the HTMT criterion, which pertains to the mean correlations of the indicators across various constructs, meets the stringent threshold of 0.85. These findings substantiate the convergent and discriminant validities of our measurement instruments.

### 3.2. Structural Model Assessment

**Figure 2:**
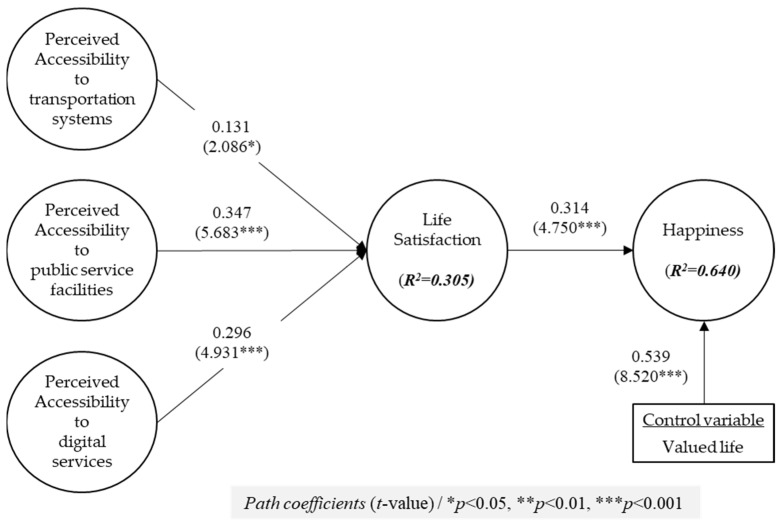
Results of path analysis.

This research utilized the Partial Least Squares (PLSs) bootstrapping methodology with a total of 5000 subsample iterations [85]. Figure 2 delineates the path coefficients and elucidates the variances associated with the research model. The values of explained variances (*R*^2^) value in Figure 2 signify that the structural model employed in this study explained 30.5% of the variance in satisfaction and 64.0% of the variance in happiness.

Regarding the impacts of perceived accessibility to living infrastructure on satisfaction, the results of this study showed that perceived accessibility to transportation systems significantly influenced satisfaction (β = 0.131, *p* < 0.05), and perceived accessibility to public service facilities affected satisfaction (β = 0.347, *p* < 0.001). Also, the results showed that perceived accessibility to digital services had a significant positive relationship with satisfaction (β = 0.296, *p* < 0.001). For the relationship between satisfaction and happiness, satisfaction had a significant positive effect on happiness (β = 0.314, *p* < 0.001). In addition, as a control variable, valued life positively affected happiness (β = 0.539, *p* < 0.001). Table 4 encapsulates the findings derived from the empirical testing of the proposed hypotheses.

## 4. Discussion

This study attempted to explain the role of perceived accessibility to living infrastructure in enhancing life satisfaction and total happiness among older adults. Living infrastructure encompasses accessibility to transportation systems, accessibility to public service facilities, and accessibility to digital services. Consistent with expectations, the findings underscore the significance of accessibility to transportation systems, public service facilities, and digital services in augmenting life satisfaction among older adults. Moreover, the explanatory capacity of accessibility to public service facilities in relation to life satisfaction was found to surpass that of other forms of accessibility.

This study has several research implications for the discipline of geriatrics. First, through the theory of motility [39], we have enlarged the scope of accessibility from a transportation-based perspective to include accessibility to residential infrastructure, which in turn has implications for life satisfaction and social participation among older adults. Second, there is a lack of emphasis on the subjective perspective within geriatric research regarding accessibility and its related social consequences, including social participation. By means of the perceived accessibility scale [43], we established metrics that signify perceived accessibility to living infrastructure. Third, we also found that higher levels of perceived accessibility to digital services were associated with increased life satisfaction among older adults. These findings are consistent with the existing body of the literature that underscores the numerous advantages of digital service use among older adults. Thus, ensuring that digital services access is equitable and providing customized support for older adults with varying digital literacy levels is imperative. While our results underscore the beneficial effects of perceived accessibility to digital services, it is vital to emphasize the importance of educational initiatives for those older individuals who are reluctant to engage with digital technologies due to experiences of ageism, thereby fostering their social engagement.

The study presents considerable implications for officials and social service professionals concerning the geriatric population. First, the findings of this study suggest that improving the physical environment of transport systems can enhance perceived accessibility and, consequently, life satisfaction among older adults. By addressing both subjective and objective barriers to transportation, policymakers have the opportunity to improve the holistic well-being of older individuals by implementing a comprehensive strategy that considers the varied needs and experiences of this demographic. Second, perceived accessibility to public service facilities is a multifaceted determinant of life satisfaction among older adults. It involves not only the physical proximity and ease of access but also the perceived quality, value, and usability of services. Enhancing these perceptions through improved service delivery, equitable access, and community engagement can significantly boost life satisfaction. Policymakers should focus on creating age-friendly environments that address both the objective and subjective aspects of service accessibility to improve the well-being of older adults. Finally, perceived accessibility to digital services positively influences life satisfaction among older adults, primarily through enhanced digital skills, social engagement, and perceived usefulness of digital technologies. However, challenges such as the lack of digital literacy and discomfort in using digital devices can negatively affect life satisfaction among older adults. Thus, enhancing digital literacy and ensuring that digital services are accessible and user-friendly for older adults is crucial for maximizing these benefits. Efforts to improve digital literacy and promote social participation can further enhance life satisfaction among adults, suggesting a need for comprehensive policies and programs that support digital inclusion and active aging.

## 5. Conclusions

The study demonstrated that perceived accessibility to living infrastructure is an essential factor for enhancing life satisfaction and happiness among older adults. The perceived accessibility to living infrastructure includes perceived accessibility to transportation systems, perceived accessibility to public service facilities, and perceived accessibility to digital services. The results show that these three components of perceived accessibility of living infrastructure serves as a more significant determinant of positive feelings among older adults. 

However, the study presented several limitations that should be factored into future research. First, this study conducted an online survey aimed at older adults. This methodological approach may lead to biases associated with under-coverage, as it neglects the older adult demographic, which lacks the capability to engage with online platforms. Thus, future studies should include older adults who are currently excluded from digital interactions. Online convenience samples exhibit significant sampling biases. Thus, future studies should incorporate mixed methods, including face-to-face interviews, to capture a more representative sample of older adults. Second, the participants for this study are individuals situated in South Korea. Generalization limitations arise from cultural, social, and contextual differences, which may affect the applicability of findings from one country to populations in different countries. Older adults in modern Korea often hold the perception that the idealized concept of ‘the good life’, characterized by reverence and authority within extended families, was universally experienced by the elderly in traditional Korea. It could lead to unique characteristics in need of care, such as their economic and housing conditions, family support, social relationships and engagement, and access to aged care services. Thus, it is essential that further research be executed with a diversified sample population spanning multiple countries to enhance the generalizability of the results. Third, the study employed cross-sectional data to examine the proposed hypotheses, which pose significant difficulties in clarifying a causal relationship between environmental factors and perceived accessibility to living infrastructure. A prospective study should consider longitudinal methodologies or alternative research designs, such as the field study approach, to validate the findings of the study. Finally, it is of paramount importance to examine the determinants that influence the perceived accessibility to living infrastructure. Even though mobility impairments pertaining to health pose a substantial issue for older adults [86], this research overlooked critical aspects such as physical inactivity, obesity, and physical disabilities, which are interconnected with limited access to living environments. Therefore, future studies should encompass a broader array of potential determinants, including objective metrics such as distance, ease of access, proximity, and health-related factors. 

## Figures and Tables

**Table 1:** Demographic characteristics of the sample.

Items	Category	Frequency	Ratio (%)
Gender	Female	105	52.5
	Male	95	47.5
Age	65~69	55	27.5
	70~74	95	47.5
	Above 75	50	25.0
Education level	High school or below	90	45.0
	Bachelor’s degree	94	47.0
	Graduate school or above	16	8.0
Average monthly income (KRW)	Below 2,000,000	11	5.5
	2,000,000–2,999,999	53	26.5
	3,000,000–3,999,999	101	50.5
	4,000,000–4,999,999	31	15.5
	Above 5,000,000	4	2.0
Residential area	Seoul City	59	29.5
	Seoul Metropolitan Area	78	39.0
	Other Metropolitan City	33	16.5
	Other areas	30	15.0

**Table 2:** Reliability of constructs.

Constructs	Mean (SD)	Cronbach’s Alpha	Composite Reliability	Average Variance Extracted (AVE)
Perceived accessibility to transportation systems	3.257 (0.898)	0.702	0.834	0.626
Perceived accessibility to public service facilities	3.250 (0.833)	0.879	0.912	0.675
Perceived accessibility to digital services	3.731 (0.849)	0.899	0.920	0.623
Satisfaction	3.035 (0.768)	0.895	0.923	0.706
Happiness	3.448 (0.776)	0.834	0.923	0.858
Valued life	3.185 (0.758)	0.863	0.906	0.708

**Table 3:** Correlation matrix and AVEs.

Constructs	ATS	APS	ADS	SAT	HAP	VAL
Perceived accessibility to transportation systems	**0.791**					
Perceived accessibility to public service facilities	0.301	**0.821**				
Perceived accessibility to digital services	0.163	0.192	**0.789**			
Satisfaction	0.284	0.443	0.384	**0.840**		
Happiness	0.116	0.238	0.265	0.714	**0.926**	
Valued life	0.146	0.271	0.213	0.741	0.741	**0.841**

Legends: ATS = perceived accessibility to transportation systems, APS = perceived accessibility to public service facilities, ADS = perceived accessibility to digital services, SAT = satisfaction, HAP = happiness, and VAL = valued life. Figures along the diagonal in bold are values of the squared root of the AVE.

**Table 4:** Summary of hypotheses testing.

Hypothesis	Path Coefficient	*t*-Value	*p*-Value	Results
Perceived Accessibility to transportation systems → Satisfaction	0.131	2.086	0.019	Supported
Perceived Accessibility to public service facilities → Satisfaction	0.347	5.683	0.000	Supported
Perceived Accessibility to digital services → Satisfaction	0.296	4.931	0.000	Supported
Satisfaction → Happiness	0.314	4.750	0.000	Supported
Valued life → Happiness	0.539	8.520	0.000	Supported

## Data Availability

The data that support the findings of this study are available from the authors upon reasonable request.

## Appendix A

**Table A1:** Measurement items.

Constructs	Items	Items	Sources
Perceived Accessibility to transportation systems	Q48	It is possible to do daily activities with the subway.	[43,72]
	Q49	It is possible to do daily activities with the bus.	
	Q50	It is possible to do daily activities with the taxi.	
Perceived Accessibility to public service facilities	Q52	Public administrative services are easily accessible.	[73,74]
	Q53	Health services are easily accessible.	
	Q54	Cultural welfare centers are easily accessible.	
	Q55	Essential facilities (including grocery stores, financial institutions, etc.) are easily accessible.	
	Q56	Educational facilities are easily accessible.	
Perceived Accessibility to digital services	Q57	It is easy for me to use public transit systems through digital resources.	[43,72]
	Q58	It is easy for me to access traffic-related information through digital resources.	
	Q59	It is easy for me to use public agency services through digital resources.	
	Q60	It is easy for me to access health-related information and services through digital resources.	
	Q61	It is easy for me to access culture-related information and services through digital resources.	
	Q62	It is easy for me to conduct banking transactions through digital resources.	
	Q63	It is easy for me to access educational information and services through digital resources.	
Satisfaction	Q15	In most ways my life is close to my ideal.	[75]
	Q16	The conditions of my life are excellent.	
	Q17	I am satisfied with my life.	
	Q18	So far I have gotten the important things I want in life.	
	Q19	If I could live my life over, I would change almost nothing.	
Happiness	Q20	In general, I consider myself: 1 (not a very happy person) to 5 (a very happy person)	[76]
	Q21	Compared to most of my peers, I consider myself: 1 (less happy) to 5 (more happy)	
Valued life	Q22	My life is worthwhile.	[70]
	Q23	My life is significant.	
	Q24	I really value my life.	
	Q25	I hold my own life in high regard.	

## Appendix B

**Table A2:** Collinearity statistics (VIF).

Construct	ATS	APS	ADS	SAT	HAP	VAL
Perceived Accessibility to transportation systems				1.114		
Perceived Accessibility to public service facilities				1.125		
Perceived Accessibility to digital services				1.052		
Satisfaction					2.220	
Happiness						
Valued life					2.220	

Legends: ATS = perceived accessibility to transportation systems, APS = perceived accessibility to public service facilities, ADS = perceived accessibility to digital services, SAT = satisfaction, HAP = happiness, and VAL = valued life.

## Appendix C

**Table A3:** Item factor loadings and cross-loadings.

Construct	Item	ATS	APS	ADS	SAT	HAP	VAL
Perceived accessibility to transportation systems	Q48	0.734	0.209	0.155	0.194	0.084	0.132
	Q49	0.837	0.189	0.137	0.220	0.045	0.122
	Q50	0.799	0.304	0.104	0.253	0.138	0.097
Perceived accessibility to public service facilities	Q52	0.313	0.725	0.208	0.296	0.149	0.154
	Q53	0.313	0.827	0.180	0.324	0.200	0.196
	Q54	0.206	0.881	0.151	0.449	0.211	0.252
	Q55	0.403	0.826	0.170	0.373	0.235	0.276
	Q56	0.198	0.840	0.097	0.352	0.173	0.216
Perceived accessibility to digital services	Q57	0.055	0.169	0.746	0.288	0.177	0.122
	Q58	0.221	0.139	0.772	0.259	0.167	0.131
	Q59	0.198	0.126	0.803	0.320	0.262	0.217
	Q60	0.054	0.120	0.779	0.370	0.262	0.229
	Q61	0.092	0.130	0.811	0.246	0.154	0.081
	Q62	0.098	0.202	0.827	0.307	0.203	0.175
	Q63	0.174	0.180	0.783	0.297	0.205	0.183
Satisfaction	Q15	0.270	0.409	0.320	0.833	0.517	0.560
	Q16	0.241	0.385	0.394	0.869	0.642	0.639
	Q17	0.218	0.343	0.380	0.885	0.666	0.672
	Q18	0.307	0.412	0.292	0.845	0.559	0.652
	Q19	0.170	0.313	0.208	0.763	0.609	0.585
Happiness	Q20	0.103	0.242	0.227	0.687	0.930	0.724
	Q21	0.080	0.197	0.265	0.633	0.922	0.705
Valued life	Q22	0.087	0.276	0.218	0.685	0.789	0.867
	Q23	0.086	0.238	0.077	0.595	0.528	0.809
	Q24	0.107	0.160	0.196	0.550	0.585	0.843
	Q25	0.226	0.224	0.204	0.646	0.644	0.846

Legends: ATS = perceived accessibility to transportation systems, APS = perceived accessibility to public service facilities, ADS = perceived accessibility to digital services, SAT = satisfaction, HAP = happiness, and VAL = valued life.

## Appendix D

**Table A4:** Heterotrait–monotrait ratio (HTMT).

Construct	ATS	APS	ADS	SAT	HAP	VAL
Perceived Accessibility to transportation systems						
Perceived Accessibility to public service facilities	0.385					
Perceived Accessibility to digital services	0.210	0.221				
Satisfaction	0.353	0.493	0.416			
Happiness	0.147	0.275	0.299	0.825		
Valued life	0.190	0.301	0.229	0.836	0.841	

Legends: ATS = perceived accessibility to transportation systems, APS = perceived accessibility to public service facilities, ADS = perceived accessibility to digital services, SAT = satisfaction, HAP = happiness, and VAL = valued life.
